# The β-cyclodextrin/benzene complex and its hydrogen bonds – a theoretical study using molecular dynamics, quantum mechanics and COSMO-RS

**DOI:** 10.3762/bjoc.9.15

**Published:** 2013-01-18

**Authors:** Jutta Erika Helga Köhler, Nicole Grczelschak-Mick

**Affiliations:** 1 Wacker Chemie AG, Consortium für elektrochemische Industrie, Zielstattstrasse 20, D-81379 München, Germany

**Keywords:** AM1, benzene, COSMO-RS, cyclodextrin, hydrogen bonds, inclusion complex, molecular dynamics, quantum mechanics

## Abstract

Four highly ordered hydrogen-bonded models of β-cyclodextrin (β-CD) and its inclusion complex with benzene were investigated by three different theoretical methods: classical quantum mechanics (QM) on AM1 and on the BP/TZVP-DISP3 level of approximation, and thirdly by classical molecular dynamics simulations (MD) at different temperatures (120 K and 273 to 300 K). The hydrogen bonds at the larger O2/O3 rim of empty β-CDs prefer the right-hand orientation, e.g., O3-H^…^O2-H in the same glucose unit and bifurcated towards ^…^O4 and O3 of the next glucose unit on the right side. On AM1 level the complex energy was −2.75 kcal mol^−1^ when the benzene molecule was located parallel inside the β-CD cavity and −2.46 kcal mol^−1^ when it was positioned vertically. The AM1 HOMO/LUMO gap of the empty β-CD with about 12 eV is lowered to about 10 eV in the complex, in agreement with data from the literature. AM1 IR spectra displayed a splitting of the O–H frequencies of cyclodextrin upon complex formation. At the BP/TZVP-DISP3 level the parallel and vertical positions from the starting structures converged to a structure where benzene assumes a more oblique position (−20.16 kcal mol^−1^ and −20.22 kcal mol^−1^, resp.) as was reported in the literature. The character of the COSMO-RS σ-surface of β-CD was much more hydrophobic on its O6 rim than on its O2/O3 side when all hydrogen bonds were arranged in a concerted mode.

This static QM picture of the β-CD/benzene complex at 0 K was extended by MD simulations. At 120 K benzene was mobile but always stayed inside the cavity of β-CD. The trajectories at 273, 280, 290 and 300 K certainly no longer displayed the highly ordered hydrogen bonds of β-CD and benzene occupied many different positions inside the cavity, before it left the β-CD finally at its O2/O3 side.

## Introduction

Cyclodextrins (CD) are a family of conical shaped cyclic oligosacharides consisting of 6–8 (or up to 10) glucopyranose units linked by α-(1→4) glycosidic bonds. At the narrower rim of the truncated cone (O6 side) there is one primary hydroxy group per glucose unit whereas at the wider rim there are two secondary hydroxy groups (O2/O3 side) [1]. Cyclodextrins have many possibilities of forming hydrogen bonds [2]: firstly, cyclodextrin monomers form some intramolecular hydrogen bonds or closed rings of hydrogen bonds at both rims of the cone; secondly, they form intermolecular hydrogen bonds with water molecules in aqueous solution or with included guest molecules that fit into their cavity. This fit is extremely specific: the orientation of one and the same guest molecule inside the cyclodextrin cavity may be opposite in solution and in crystalline state, as was found for 4-fluorophenol in α-CD [3]. The reactivity of aromatic guest molecules, radicals or excited states, was found to be altered because of complex formation with cyclodextrins [4].

Cyclodextrins form three types of dimers, O2/O3 to O2/O3, O2/O3 to O6, and O6 to O6. They can also associate to extended stacks in crystalline state or solution, with guest and solvent molecules located inside the cavities, between dimers, or in the channels next to the stacks [5]. The influence of cyclodextrin on a broad variety of molecules was reported: from changes of α-/β-sheet ratio upon complex formation of cyclodextrins with side chains of proteins [6], to size control of the electrostatic self assembly of nanoparticles [7]. Also a pH-controlled release of many cyclodextrins in long stacks on polymers, such as polyrotaxanes, combined with mesoporous silica particles was observed [8].

**Figure 1:**
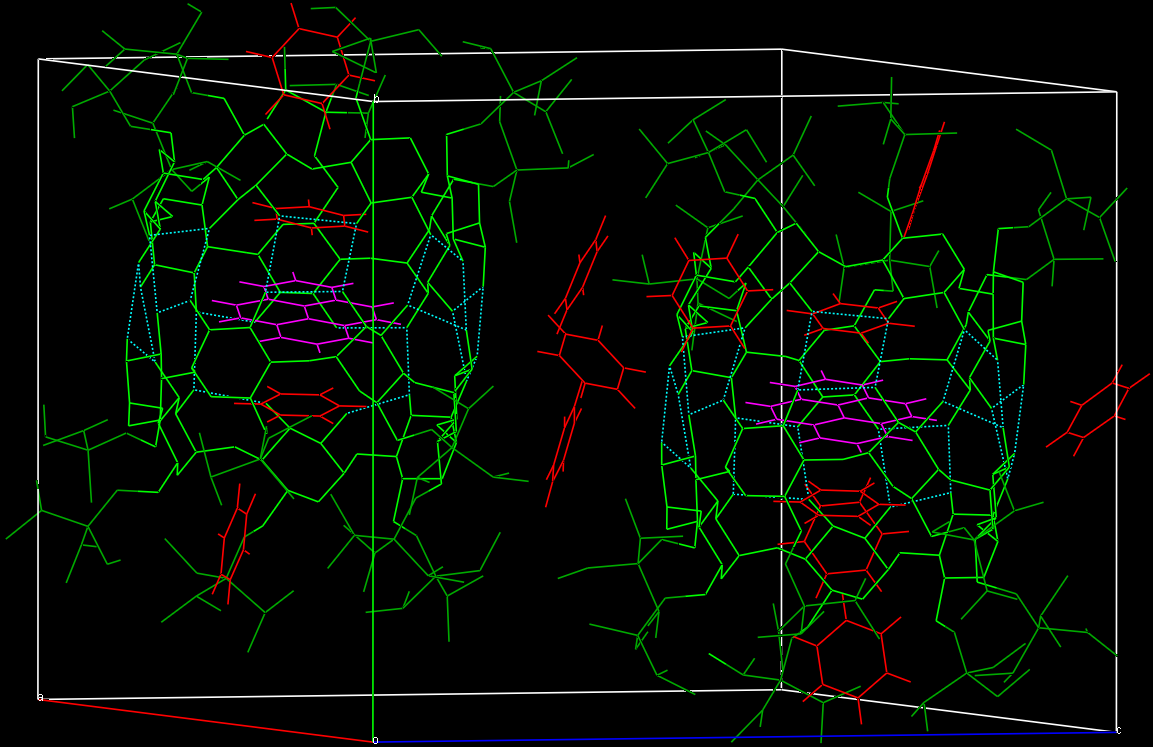
Crystal structure of heptakis(6-*O*-triisopropylsilyl)-β-cyclodextrin benzene pyrene solvate; [C_105_H_210_O_35_Si_7,0.5_(C_16_H_10_),_3.5_(C_6_H_6_)], taken from The Cambridge Crystallographic Data Centre CCDC [9]. Colour code: benzene: red; pyrene: magenta; cyclodextrin corpus: light green; cyclodextrin side chains: dark green; hydrogen bonds: light blue, dotted lines.

Complex formation with solvent molecules can take place at the same time in different positions: the crystal structure of heptakis(6-*O*-triisopropylsilyl)-β-CD benzene pyrene solvate is a nice example for competing solvents that intercalate in the cavity of one β-CD (benzene), between β-CD dimers (pyrene), and in channels outside β-CDs (benzene). The β-CD dimers are connected with hydrogen bonds on their O2/O3 sides (Figure 1).

Investigations of such “flat energy hypersurfaces” due to hydrogen bonds and noncovalent interactions demand several theoretical methods to capture the entire network of forces on which they rely. Molecular dynamics simulations displayed different hydrogen bond patterns of cyclodextrins in crystal and in solution and confirmed the experimental findings of soluble cyclodextrin complexes of cholesterol type versus precipitates of *cis*/*trans*-cyclohexadecenone//CDs [10–12]. MD with λ-dynamics and calculation of the solvation-free-energy differences was used to distinguish amylose helices from cellulose sheets by analysing the different reactivity of oxygen atoms O2, O3 and O6 of the sugar units with and without methylation, in line with experimental data [13]. Cyclodextrin-complex formation with substituted benzenes shows multiple topologies/configurations (guest up/down) in MD with λ-dynamics [14]. The association constant *K*_a_ for α- and β-CD inclusion complexes with several benzene derivatives was investigated by a genetic algorithm [15] and neuronal networks [16]. An overview of quantum mechanical methods to study cyclodextrin chemistry is given in [17]. The COSMO-RS solvation model [18] allows for calculations of several thermodynamic properties [19] once the polarisation charge surface of molecules has been determined by DFT calculations. With the current implementation of COSMO-RS, hydrogen-bond energies are predicted within 0.36 kcal mol^−1^ relative to QM dimer calculations [20].

The necessity to understand the function of cyclodextrins in all details arises from their many applications in industry [21], agriculture, food [22] and health care [23]. Cyclodextrins and their complexes are produced in large industrial processes [24–26]. Our intention for this study was to find a “modular, robust molecular-modelling workflow” for how to analyse the formation of cyclodextrin complexes for many guest molecules in general, before extensive experimental work is carried out. Lemon grass oil [27] or retinol [23] can serve as typical molecular examples of the “practical industrial workbench”. To reach reasonable structures of these complexes we need to construct several models because of (a) isomers, (b) *n*:*m* guest/host stoichiometry, (c) preferred cyclodextrin cavity size, etc. We start here with the first step, the empty β-CDs, their subtle but strongly influential hydrogen bonds, and their complex formation with the simplest aromatic molecule of highest symmetry without substituents, i.e., benzene [28].

## Methods

While calculating several cyclodextrin complexes with molecular dynamics [29], semi-empirical AM1, classical quantum mechanics [30] and COSMO-RS (Turbomole) [31], we observed that the orientation of all hydrogen bonds of the cyclodextrins already played a crucial role when the initial structures were constructed. It was not sufficient just to take the “normal route” of using the energetically lowest-lying structures from MD trajectories, and then proceed to AM1 and further to QM optimizations; on the contrary, these structures turned out not to be the best ones possible. The best ones in QM finally were those that had hydrogen bonds oriented as symmetrically as possible. Therefore, we started with such manually constructed models and their AM1 optimisations.

Step 1: Our four β-CD models are conformers; all the hydrogen bonds of each rim are oriented in the same direction. We named them BCDO23lO6l, BCDO23rO6l, BCDO23lO6r and BCDO23rO6r, referring to their manually constructed hydrogen-bond orientations when looking either at their O2/O3 or O6 rim (l = left hand, r = right hand orientation) by using GaussView [30]. Each model was optimised until convergence was reached “AM1 (opt=calcall, verytight)” by using the Gaussian9W program [30]. Molecular properties, such as energy, dipole moment, HOMO/LUMO molecular orbitals and IR spectral data, were analysed with GaussView.

Step 2: The four AM1 optimised β-CD models were used as starting structures in our QM calculations by using Turbomole v. 6.4 with COSMO-RS [31]. Two models were calculated for each structure, i.e., the structure in vacuo (.energy files) and the COSMO-RS structure (.cosmo files), the latter being optimised in a dielectric field with the dielectric constant of water (the value of the dielectric constant is a variable in the COSMO-RS program and can be chosen by the user). Up to now it was possible to calculate molecular structures with COSMO-RS on DMOL, BP/SVP or BP/TZVP level of approximation. Our trial to optimise the β-CD complexes with BP/TZVP failed: not always but in several cases, here, for example, the benzene guest was expelled for the BCDbenzeneO23lO6r conformers. Now, however, the current version of Turbomole allows the calculation of more detailed energy hypersurfaces especially for hydrogen bonds (keyword BP-TZVP-DISP3) since Grimme’s dispersion corrections for nonbonded interactions were implemented recently [32]. The current release of COSMO-RS C30_1201 allows, for the first time, a more detailed σ-surface to be calculated (BP/TZVPD-FINE), but for the time being as single points (SP) only. COSMO-RS structures are saved in COSMO databanks to perform graphical and thermodynamic analysis with COSMOthermX [19]. In this study we used the BP/TZVP-DISP3 method to calculate the β-CD models, and with this method no benzene molecule was expelled from the β-CD cavity any more.

Step 3: Starting from the neutron diffraction study of β-CD [33] we constructed one model named “BCDcry” of one β-CD by adding hydrogen atoms using the Visualizer of Materials Studio v. 5.5 [29]. Additionally, we used the four empty β-CDs of the in vacuo runs (BP/TZVP optimised .energy files) and the two best structures from the AM1 optimisations, BCDO23rO6l/benzene parallel and vertical. With Materials Studio molecular dynamics (Forcite plus program and Compass force field) we simulated their trajectories at 300 K. In detail: After force-field geometry optimisations the structures were heated up each from 0 to 3 K, to 100 K, to 200 K, and to 300 K. Finally at 300 K a trajectory of 6000 ps length was calculated using an integration step of 1 fs. From the last 2000 ps of the trajectories the averaged total energy *E*_tot_ <4000–6000>, averaged hydrogen-bond energy *E*_HB_ <4000–6000>, and the averaged hydrogen-bond distance and angle were calculated.

Next, the energetically two best optimised AM1 structures (BCDO23rO6l with benzene included in parallel and vertical positions) were used as starting structures and simulated at 300, 290, 280, and 273 K for 500 ps each, and their release of the guest was analysed from these trajectories.

## Results and Discussion

### AM1 Calculations

#### The four conformers of empty β-CD

The BCDO23rO6l structure turned out to be the lowest energy conformer, also having the lowest dipole moment of only 0.5618 Debye (dipole-moment components *x* = 0.0; *y* = 0.0; −*z* = 0.5618) pointing from the O6 rim towards the O23 rim of the cone. This structure is so symmetric that all its Mulliken partial atomic charges on all types of oxygen atoms were identical, e.g., equal to each average number (Table 1). Second, with about 6 kcal mol^−1^ above, was conformer BCDO23rO6r. This oval structure has the highest dipole moment of 2.5639 D (*x* = +0.0268; *y* = +0.0236; *z* = −2.5637) and its O6 atomic charges display a range from −0.340 to −0.349 (average −0.344), but all O2 atoms still have the same charge of 0.325, and O3 and O4 oxygen charges vary very little (−0.337 to −0.338 and −0.300 to −0.301, respectively). The third conformer BCDO23lO6l, lying about 7 kcal mol^−1^ above, was again total symmetric as shown by its dipole moment components of *x* = 0.0; *y* = 0.0; *z* = −1.0687 and also with respect to all its oxygen charge values. The fourth conformer, BCDO23lO6r, had the highest energy of about 12.7 kcal mol^−1^ compared to the ground state BCDO23rO6l and the second largest dipole value of 2.4223 D (*x* = +0.5806; *y* = −0.0872; *z* = −2.3501). Its O6 oxygen atoms display a broad range of Mulliken partial charges from −0.338 to −0.360, although their average hardly differs from the average values of the other O6 types (see Table 1); O2 and O3 have small ranges (−0.333 to −0.335 and −0.345 to −0.347) but some differences can be observed in the O4 values, i.e., −0.288 to −0.296. These subtle differences were just given as a few examples here in the text (e.g., they are not given in Table 1) since they may be of importance during guest inclusion or reactivity. The Mulliken partial atomic charges of the benzene molecule in *D*_6_*_h_* symmetry were C: −0.130 and H: +0.130.

**Table 1:** AM1 results for the four empty β-CD conformers. AM1 optimised energies [a.u.], relative energies [kcal mol^−1^], dipole moments [D], LUMO/HOMO energies [a.u.] and gap [eV], and averaged Mulliken partial atomic charges of oxygen atoms. Benzene molecule with *D*_6_*_h_* symmetry in vacuo. Δ*E* is the energy of each conformer with respect to the lowest conformer in this table, which is O23rO6l (bold).

AM1 β-CD empty^a^	abs. *E* [a.u.]	Δ*E* [kcal mol^−1^]	μ [D]	LUMO/HOMO [a.u.]; gap [eV]	O2 atomic charge	O3 atomic charge	O4 atomic charge	O6 atomic charge

O23lO6l	−2.653658	7.06	1.0687	+0.06271 −0.38469 12.17	−0.335	−0.347	−0.291	−0.348
O23lO6r	−2.644708	12.68	2.4223	+0.06083 −0.38424 12.11	−0.334	−0.346	−0.293	−0.346
**O23rO6l**	**−2.664914**	**0.00**	**0.5618**	**+0.06072** **−0.38650** **12.17**	**−0.325**	**−0.337**	**−0.297**	−**0.348**
O23rO6r	−2.655373	5.99	2.5639	+0.05835 −0.38786 12.14	−0.325	−0.337	−0.301	−0.344
benzene^b^	+0.034953		0.0000	+0.02039 −0.35474 10.21				

^a^All four β-CD conformers reached a RMS gradient norm of 0.00000001 [a.u.] in the geometry optimisations. ^b^Benzene reached a RMS gradient norm of 0.00000002 [a.u.].

#### The four conformers of the β-CD complexes with benzene in a parallel or vertical position

**Figure 2:**
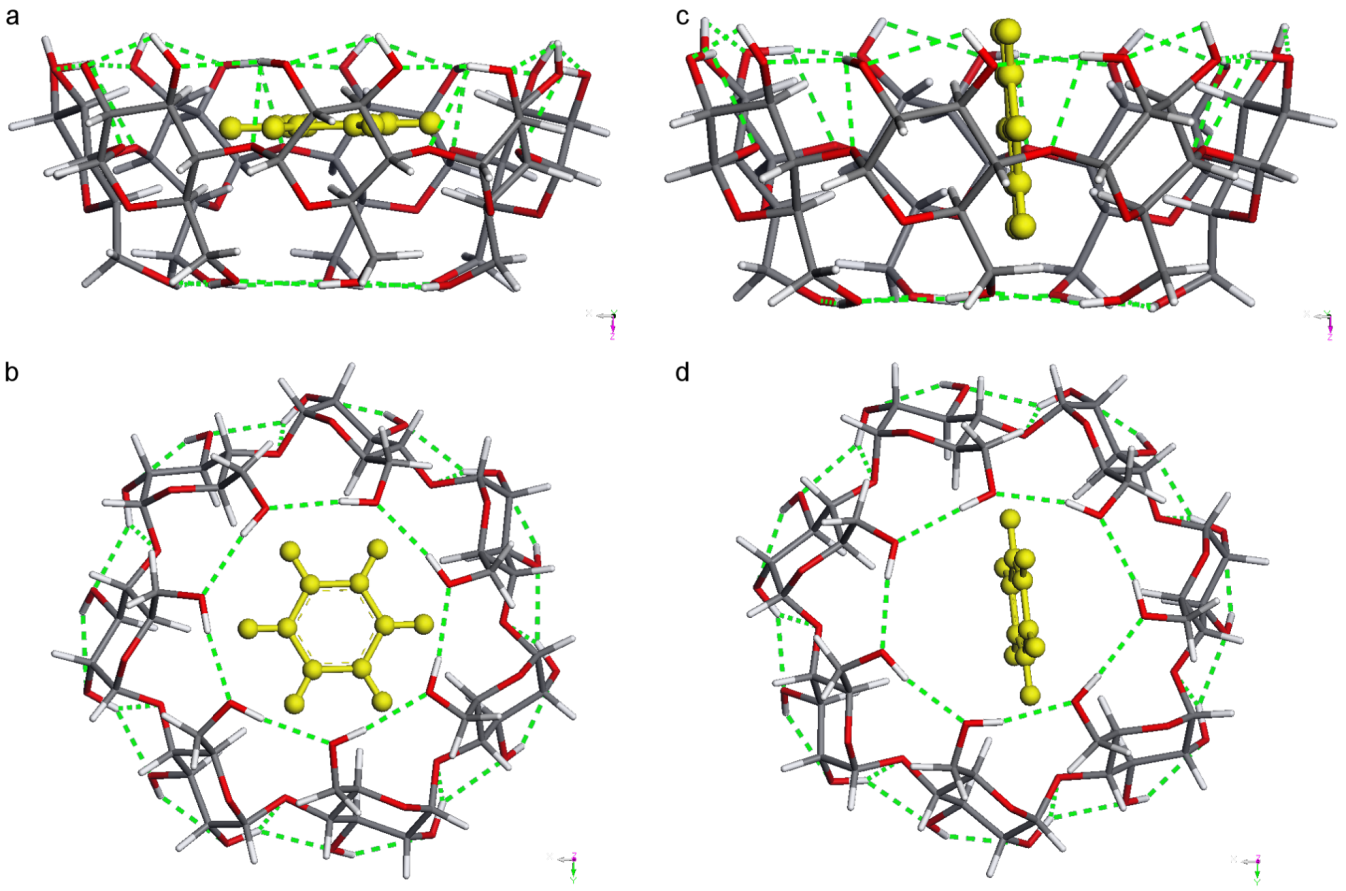
The two AM1-optimised stable conformers of BCDO23rO6l with benzene in a parallel (a and b) and vertical position (c and d). Top view from the O6 side.

All four conformers of the complexes preferred the inclusion of benzene in a parallel position (Table 2). The energetic order of the benzene complexes and their sequence of dipole moments was qualitatively the same as for the empty ones: BCDO23rO6l, BCDO23rO6r, BCDO23lO6l, and BCDO23lO6r. Benzene was slightly distorted in each cavity, as can be seen from its energy and dipole moment inside the complex. Inside the β-CD/benzene complex the benzene neither in a parallel position nor in vertical position adopted an exact central position as displayed in Figure 2.

**Table 2:** AM1 results for the four β-CD conformers with benzene placed in a parallel or vertical position. AM1 absolute energies, relative energies and dipole moments for the complexes: columns 1–3; for benzene alone: columns 4 and 5.

AM1 β-CD/benzene complex^a^	complex abs. *E* [a.u.]	complex Δ*E* [kcal mol^−1^]	complex μ [D]	benzene abs. *E* [a.u.]	benzene μ [D]

O23lO6l parallel vertical	−2.62313280 −2.62266055	7.0374744 7.3338160	0.9544 0.7084	0.03501077 0.03499769	0.0446 0.0092
O23lO6r parallel vertical	−2.61493920 −2.61452474	12.1790404 12.4391182	2.9750 1.5541	0.03499519 0.03500111	0.0174 0.0104
O23rO6l **parallel** **vertical**	**−2.63434772** **−2.63388503**	**0.00000000** **0.29034260**	**0.4399** **0.1579**	**0.03502037** **0.03499812**	**0.0543** **0.0096**
O23rO6r parallel vertical	−2.62574636 −2.62459809	5.39743941 6.11799032	2.5365 1.9127	0.03499903 0.03499288	0.0256 0.0138

^a^All four complexes reached a RMS gradient norm ≤ 3 × 10^−8^ [a.u.] in the geometry optimisations.

Our calculated AM1 HOMO/LUMO gaps of empty β-CD conformers, of benzene *D*_6_*_h_* (Table 1) and the eight complexes (Table 3) are of the same order of magnitude as the values calculated with PM3 in the literature [34] where they found for β-CD 12.44 eV, for benzene 10.15 eV and for the β-CD/benzene complex 10.15 eV. These authors found β-CD/benzene of 1:1 inclusion type in aqueous solution by experimental UV absorption spectra with various concentrations of β-CD. From their UV plots they calculated their stability constants *K*_s_ at three temperatures to be 116 L mol^−1^ at 291 K, 86 L mol^−1^ at 298 K and 68 L mol^−1^ at 303 K. According to the equation Δ*G* = −RT ln *K*_s_ they obtained the corresponding three free energy values Δ*G* of −11.5 kJ mol^−1^, −11.0 kJ mol^−1^ and −10.6 kJ mol^−1^, indicating rather weak interactions between the guest and host molecule, but still a thermodynamically exothermic reaction of complex formation.

Here, our best conformer, BCDbenzeneparallelO23rO6l, displays an energy of complex formation

[1]
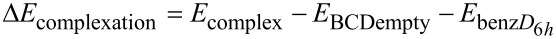


of −2.75 kcal mol^−1^ (Table 3), followed by the complex conformer BCDbenzeneverticalO23rO6l with −2.46 kcal mol^−1^. This is in very good agreement with the experimental value from [34]. All our other complex conformers have positive Δ*E*_complexation_ values, up to BCDbenzeneverticalO23lO6r with +9.69 kcal mol^−1^, and thus are endothermic, Table 3.

**Table 3:** AM1 results for the four β-CD/benzene complex conformers with benzene placed in a parallel or vertical position. LUMO/HOMO energies and band gaps: columns 1–3. Absolute energies of complex formation Δ*E*_complexation_: columns 4,5. Data of column 5 are given in kilojoules per mole (kJ mol^−1^) for easy comparison to the text above and [34].

AM1 β-CD/benzene complex	HOMO [a.u.]	LUMO [a.u.]	HOMO/LUMO gap [eV]	Δ*E*_complexation_ [kcal mol^−1^]	Δ*E*_complexation_ [kJ mol^−1^]

O23lO6l parallel vertical	−0.34409 −0.34163	0.03063 0.03241	10.196 10.178	4.29 4.58	17.93 19.17
O23lO6r parallel vertical	−0.35373 −0.34592	0.02092 0.02861	10.195 10.192	9.43 9.69	39.44 40.53
O23rO6l **parallel** **vertical**	**−0.34784** **−0.34530**	**0.02688** **0.02866**	**10.197** **10.176**	**−2.75** **−2.46**	**−11.52** **−10.30**
O23rO6r parallel vertical	−0.35776 −0.35353	0.01689 0.02046	10.195 10.177	2.65 3.37	11.07 14.08

#### IR spectra of empty β-CD conformers and their inclusion complexes

**Figure 3:**
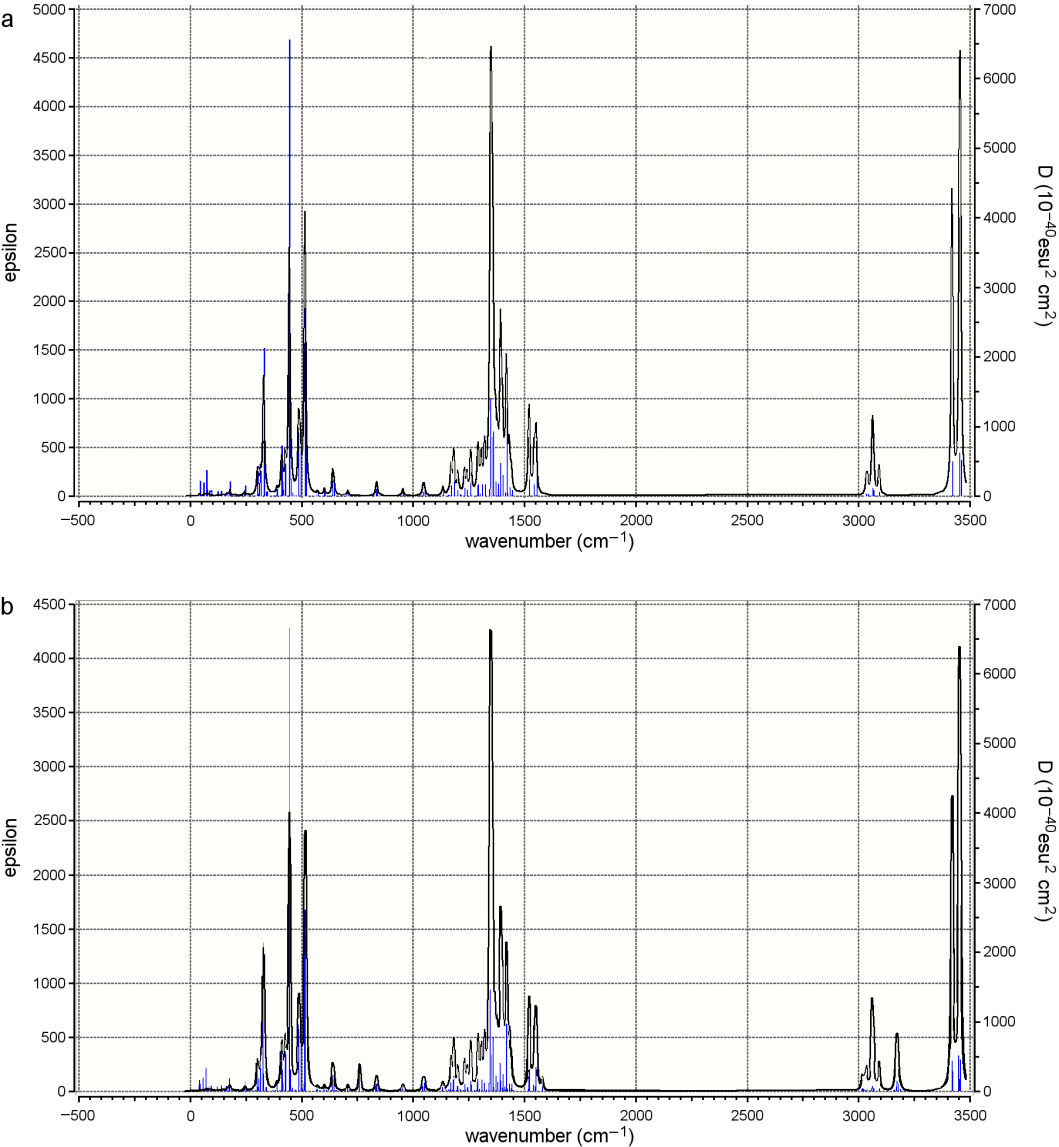
(a) AM1 calculated IR spectra of BCDO23rO6l empty and (b) the BCDO23rO6l/benzene inclusion complex with benzene in a parallel orientation.

The most important lines of the calculated AM1 IR spectra are given in Table 4. The IR bands of benzene at 744, 1145, 1579 and 3194 cm^−1^ were characteristically shifted in the parallel versus the vertical complex. Especially the 3194 cm^−1^ H–C benzene stretch was split into a range from 3150 to 3199 cm^−1^. Very interesting and easy to see was the concerted movement of all hydrogen bonds at the O23 and O6 rim: the empty β-CD’s H–O2 stretch at 3419 cm^−1^ was spread out to a range from 3414 to 3421 cm^−1^, the H–O6 stretch at 3453 cm^−1^ was spread out from 3447 to 3453 cm^−1^, and the β-CD’s H–O3 stretch at 3457 cm^−1^ from 3455 to 3456 cm^−1^ as a result of the interaction with the guest molecule. In general, the shifts were subtle; it seemed that the benzene itself was influenced very little if at all, the cyclodextrin on the contrary was substantially influenced in its frame vibrations over a wider range, its splitting most probably being caused by the “rigid benzene wheel” it had included, see Figure 3. Interestingly, the concerted hydrogen-bond movements at the rims of β-CD rims were easily identified, for example in the BCDbenzeneparallelO23rO6l complex. Here, at 445 cm^−1^, all hydrogen atoms bound to O6 atoms concertedly moved up and down the almost perfect plane of O6 oxygen atoms (marked H(O6) up/d in Table 4).

**Table 4:** AM1 results for benzene and the two stable O23rO6l β-CD conformers with benzene placed in a parallel or vertical position. All IR bands [cm^−1^] and relative intensities are listed for benzene; in the case of β-CD, single bands are included only if their relative intensity is >200 or for “very close frequency clusters” (bold)^a^.

AM1 IR freq. [cm^−1^]	rel. intensity benzene *D*_6_*_h_* sym.	rel. intensity β-CD O23rO6l empty	rel. Intensity β-CD O23rO6l parallel	rel. Intensity β-CD O23rO6l vertical	comment

443					H(O6) up/d
444		729		713	H(O6) up/d
445			741		H(O6) up/d

511				276	H(O2) up/d
513		2 × 346	286		H(O2) up/d
514				240	H(O2) up/d
516			335	230	H(O2) up/d

744	127				H–C up/d
757				122	H–C up/d
760			73		H–C up/d

1145	2 x 1.08				H–C in plane
1148				1.32	H–C in plane
1150			1.74		H–C in plane
1150			1.51		H–C in plane
1152				0.54	H–C in plane

1346				344	O4 and O5
1347		471	491		O4 and O5
1347		471	355		O4 and O5
1348				291	O4 and O5

1351		2 × 285			C-O6 frame
1356–1357			**534(3)**^a^		C-frame
1357		2 × 312			C-frame
1357–1358				**562(3)**^a^	C-frame

1418		346		338	H–C up/d
1419			339		H–C up/d

1577				14	C=C
1578			15		C=C
1579	2 × 12		15	10	C=C

3150				16	H–C benz.
3158				22	H–C benz.
3165				91	H–C benz.
3170				35	H–C benz.
3171			107		H–C benz.
3175	2 × 88		75		H–C benz.
3186			12		H–C benz.
3189				20	H–C benz.
3194					H–C benz.
3199				17	H–C benz.

3414–3421				**879(7)**^a^	H–O2 stretch
3418–3421			**861(7)**^a^		H–O2 stretch
3419		2 × 425			H–O2 stretch

3447–3455				**733(5)**^a^	H–O6 stretch
3449–3451			**1004(7)**^a^		H–O6 stretch
3453		2 × 536			H–O6 stretch

3455–3456			**563(7)**^a^		H–O3 stretch
3457		**532(3)**^a^			H–O3 stretch

3456–3461				**832(9)**^a^	H–O6/H–O3 stretch

^a^Sum of (*n*) intensities within the given frequency range for a cluster of the same type (bold).

Since our two stable β-CD/benzene complex conformers (parallel and vertical) were energetically close neighbours with only 0.29 kcal mol^−1^ energy difference calculated at 0 K, the experimental spectrum at room temperature should display all cyclodextrin absorptions, i.e., both our conformers exist at the same time.

#### Summarised result of the AM1 calculations

AM1 energy/geometry optimisations in vacuo at 0 K showed that the β-CD/benzene complex with all hydrogen bonds at the O2/O3 rim in a right-hand orientation and at the narrower O6 rim in a left-hand orientation can include benzene in a parallel (best energy) or vertical position (higher in energy by only 0.29 kcal mol^−1^); both conformers were thermodynamically allowed. The HOMO/LUMO gap of the empty β-CD with about 12 eV was lowered to about 10 eV in the complex. The β-CD/benzene inclusion complex was formed only by weak noncovalent interactions, which influence the IR lines of the β-CD most at its frame frequencies and at highly ordered concerted movements of its hydrogen bonds. The IR frequencies of benzene were influenced only marginally upon inclusion.

### COSMO-RS calculations with Turbomole

#### Quantum mechanical geometry/energy optimisations

For the four empty β-CD models, for benzene in *D*_6_*_h_* geometry, and for all eight BCD/benzene complexes, quantum mechanical geometry/energy optimisations with Turbomole and the implemented COSMO-RS method were performed at 0 K. For all starting structures the AM1 optimised geometries were used, and the results are given in Table 5 for the calculations in vacuo, and in Table 6 for the calculations in the COSMO-RS dielectric field of water (no explicit water molecules, but instead dipoles that model the dielectric field of the solvent are used by this method [18]).

**Table 5:** Optimised BP/TZVP-DISP3 energies in vacuo. Lowest energy conformer of each group in bold.

BP/TZVP-DISP3 in vacuo	*E*_abs._ [a.u.]	*E*_rel._ [a.u.]	*E*_rel._ [kcal mol^−1^]

BCDO23lO6l	−4277.4641711793	0.0004226810	0.2652365729
BCDO23lO6r	−4277.4609298546	0.0036640057	2.2992002354
**BCDO23rO6l**	**−4277.4645938603**	**0.0000000000**	**0.0000000000**
BCDO23rO6r	−4277.4611238596	0.0034700007	2.1774601643
Benzene	−232.3358156576	0.0071740456	4.5017853670
parallelO23lO6l	−4509.8320598074	0.0005758687	0.3613633618
parallelO23lO6r	−4509.8289075499	0.0037281263	2.3394365033
parallelO23rO6l	−4509.8325394613	0.0000962148	0.0603757489
parallelO23rO6r	−4509.8291543743	0.0034813018	2.1845516926
verticalO23lO6l	−4509.8321043279	0.0005313482	0.3334263276
verticalO23lO6r	−4509.8289158257	0.0037198504	2.3342433180
**verticalO23rO6l**	**−4509.8326356761**	**0.0000000000**	**0.0000000000**
verticalO23rO6r	−4509.8291521426	0.0034835335	2.1859520815

**Table 6:** Optimised BP/TZVP-DISP3 COSMO-RS energies in aquo. Lowest energy conformer of each group in bold.

BP/TZVP-DISP3 in aquo	*E*_abs._ [a.u.]	*E*_rel._ [a.u.]	*E*_rel._ [kcal mol^−1^]

BCDO23lO6l	−4277.5397933200	0.0044818408	2.8123999202
BCDO23lO6r	−4277.5390925770	0.0051825838	3.2521231603
**BCDO23rO6l**	**−4277.5442751608**	**0.0000000000**	**0.0000000000**
BCDO23rO6r	−4277.5432219210	0.0010532398	0.6609185069
Benzene	−232.3408624099	0.0021272933	1.3348978187
parallelO23lO6l	−4509.9064609135	0.0041674010	2.6150858014
parallelO23lO6r	−4509.9056675976	0.0049607169	3.1128994621
**parallelO23rO6l**	**−4509.9106283145**	**0.0000000000**	**0.0000000000**
parallelO23rO6r	−4509.9096646704	0.0009636441	0.6046963092
verticalO23lO6l	−4509.9004752425	0.0101530720	6.3711542109
verticalO23lO6r	−4509.9055329919	0.0050953226	3.1973658847
verticalO23rO6l	−4509.9106053475	0.0000229670	0.0144120224
verticalO23rO6r	−4509.9095882112	0.0010401033	0.6526752221

**Figure 4:**
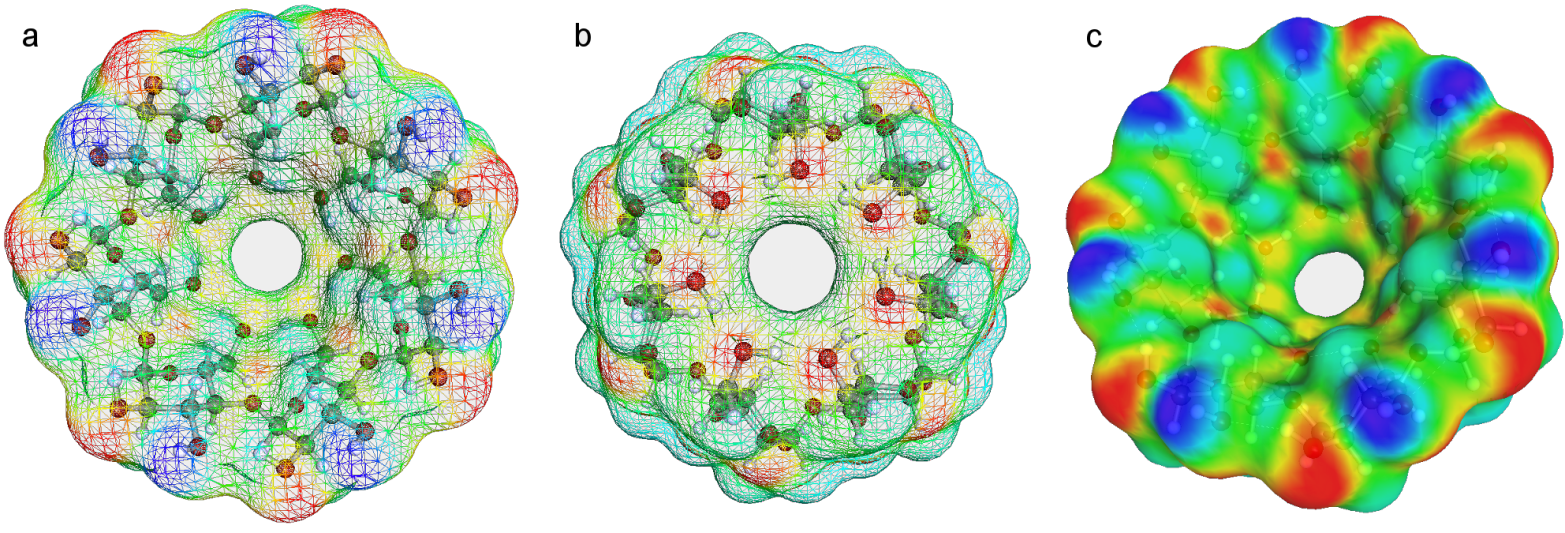
The lowest energy empty conformer BCDO23rO6l, COSMO-RS structure optimised with BP/TZVP-DISP3 (solvent = water), σ-surface with charge code: oxygen red, hydrogen blue, hydrophobic area yellow and green. View a: from O2/O3 side, view b: from O6 side, view c: from O2/O3 side.

The empty BCDO23rO6l model is still the lowest energy conformer among the four in vacuo (Table 5), but now the BCDO23lO6l is second and energetically close (0.26 kcal mol^−1^), followed by BCDO23rO6r with 2.17 kcal mol^−1^ and BCDO23lO6r with 2.3 kcal mol^−1^ higher. In aquo (Table 6) the empty hydrogen bond model BCDO23rO6l was again the most preferred (Figure 4).

Although all optimisations were started from the AM1-optimised parallel and vertical geometries (from which the naming stems), the BP/TZVP-DISP3 method with its enhanced description of dispersion forces led in general to structures in which the benzene molecule adopts a more oblique position [14,20,32]. The whole energy hypersurface of β-CD complexes is rather flat, and therefore it is difficult to identify the many local minima [17]. Therefore, geometry optimisations of β-CD complexes still remain a multiple-minimum problem in general. The COSMO-RS method combines these identified multiple-minima structures as a set of conformers in a databank (which is updated principally when new minima are identified by more sensitive theoretical methods). From these sets the thermodynamic properties of the material at various temperatures can be calculated (by analytical formulas with COSMOthermX) more realistically than for one structure only [18–20]. Molecules adopt many conformers at temperatures higher than 0 K, which will be shown for the β-CD/benzene complex by MD simulations in the next section. The energetically best structure of the BCD/benzene complex in vacuo due to the BP/TZVP-DISP3 method resulted from the BCDO23rO6l/benzenevertical starting structure. The AM1 favourite BCDO23rO6l/benzeneparallel model followed second with 0.06 kcal mol^−1^ above, and the others were higher up to a maximum of 2.34 kcal mol^−1^ (BCDO23lO6r/benzeneparallel), Table 5. All optimised BP/TZVP-DISP3 COSMO-RS energies in aquo were lower than the corresponding ones in vacuo, which is reasonable (Table 6). Here the BCDO23rO6l hydrogen-bond starting models remained the best ones after energy/geometry minimisations, for the empty β-CD and for the complex as well.

The BP/TZVP-DISP3 energies of complex formation were calculated from the data of Table 5 and Table 6 by Equation 1 and are shown in Table 7. All energies of complex formation Δ*E*_Complexation_ in vacuo and in aquo were negative, indicating that the complexes will be formed. The complex structures of the reactions with the lowest energies were BCDO23rO6l/benzeneparallel in aquo and BCDO23rO6l/benzenevertical in vacuo.

**Table 7:** BP/TZVP-DISP3 energies of complex formation in vacuo and in aquo, calculated with the absolute data of BCDO23rO6l for *E*_BCDempty_. Relative Δ*E*_Complexation_ with respect to the energetically lowest complex conformer, column 2 and column 4.

BP/TZVP-DISP3	abs. Δ*E*_Complexation_ in vacuo [kcal mol^−1^]	rel. Δ*E*_Complexation_ in vacuo [kcal mol^−1^]	abs. Δ*E*_Complexation_ in aquo [kcal mol^−1^]	rel. Δ*E*_Complexation_ in aquo [kcal mol^−1^]

parallelO23lO6l	−19.861	0.361	−13.381	2.615
parallelO23lO6r	−17.883	2.339	−12.883	3.113
**parallelO23rO6l**	−20.162	0.060	**−15.996**	**0.000**
parallelO23rO6r	−18.038	2.184	−15.391	0.605
verticalO23lO6l	−19.889	0.333	−9.625	6.371
verticalO23lO6r	−17.888	2.334	−12.798	3.198
**verticalO23rO6l**	**−20.222**	**0.000**	−15.981	0.015
verticalO23rO6r	−18.036	2.186	−15.343	0.653

**Figure 5:**
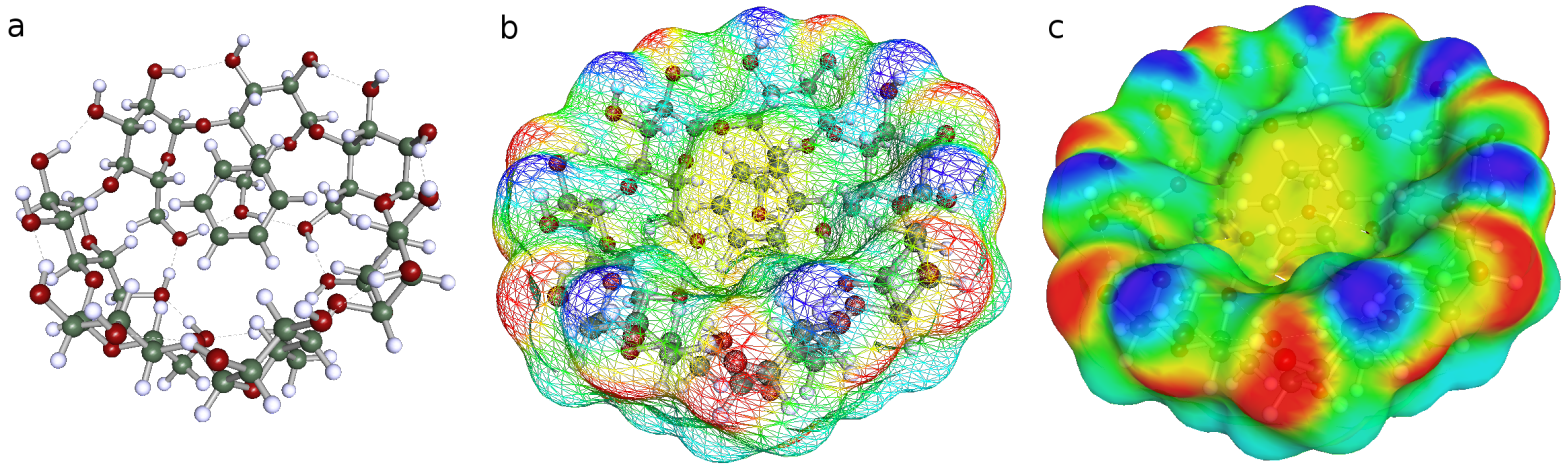
The lowest energy complex conformer BCDO23rO6l/benzene; benzene occupies an oblique position inside the cavity. COSMO-RS structure optimised with BP/TZVP-DISP3 (solvent = water), a: no surface, b,c: σ-surface with charge code: oxygen red, hydrogen blue, hydrophobic area yellow and green. All views from O2/O3 side.

Figure 4a–c and Figure 5a–c display different views of the best BCDO23rO6l empty model and the BCDO23rO6l/benzene complex with their COSMO-RS σ-surfaces. Hydrophilic areas are coloured in red (negative charge of oxygen atoms) or blue (positive charge of hydrogen atoms), which were mostly located at the O23 rim. Hydrophobic areas (coloured green/yellow) can be observed easily inside the BCDO23rO6l empty model (caused by its O6 highly ordered hydrogen-bond rim) and the whole surface of benzene while sitting inside the cyclodextrin cavity.

#### Consequences for thermodynamic analysis

**Figure 6:**
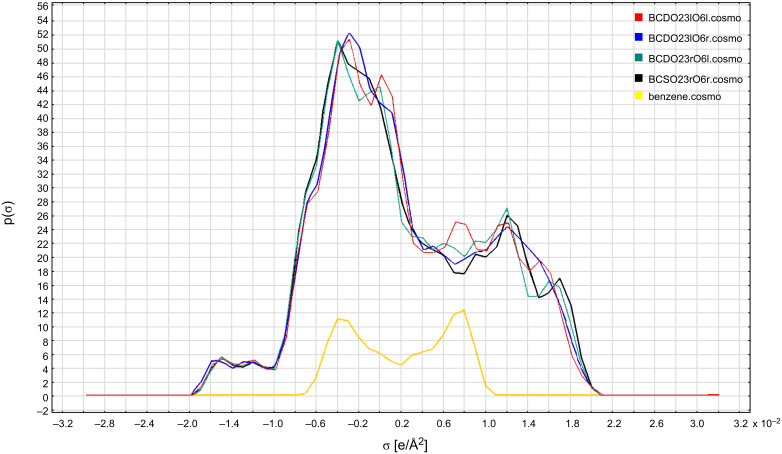
σ-Profiles of the COSMO-RS method for the four empty β-CD models and benzene (BP/TZVP-DISP3 method).

**Figure 7:**
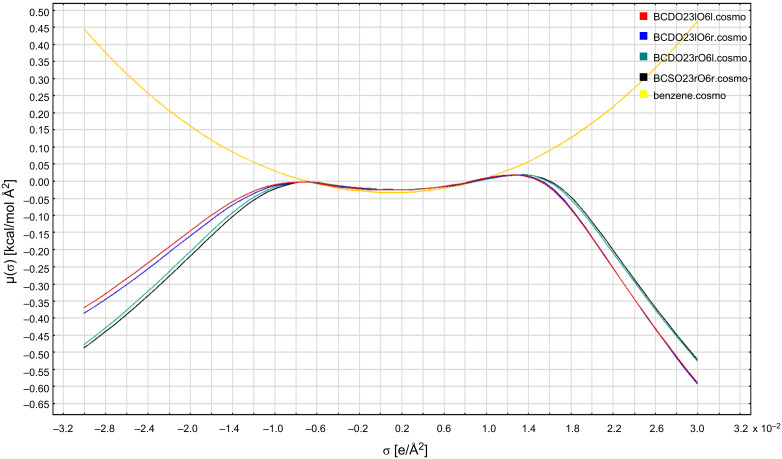
σ-Potentials of the COSMO-RS method for the four empty β-CD models and benzene (BP/TZVP-DISP3 method).

The basis of thermodynamic analysis with COSMOthermX [18–20] is the so called σ-profiles and σ-potentials of each molecule, which show the characteristic differences in the electrostatic molecular surfaces that are calculated quantum mechanically at 0 K for molecular conformers of up to 10 kcal mol^−1^ in the standard COSMO databases. These σ-profiles and σ-potentials were calculated with COSMOthermX for the empty β-CD models and are displayed in Figure 6 and Figure 7. Especially the σ-potentials (Figure 7) showed that the empty β-CD models were split into two groups. One group had the two hydrogen-bond models with all hydrogen bonds in the left-hand orientation (O23l), the other one with the two models with right-hand (O23r) orientation. This mirrored quantitatively the result of how much the two energetically preferred O23r models were closer to each other and were most different from the two O23l models, regardless of which orientation the hydrogen bonds had at the O6 rim. By the time these four empty β-CD models have been combined into one set of β-CD conformers and stored like this in the COSMO database, thermodynamic analysis with COSMOthermX will take into account these differences and give more accurate results for many material properties over a wider temperature range than with simple “one-molecule sets” [18–20].

#### Summarised result of the COSMO-RS calculations

In aquo the hydrogen-bond model BCDO23rO6l was again the most preferred, not only among the empty β-CD conformers but also as the BCDO23rO6l/benzene parallel complex model. According to the quantum mechanical COSMO-RS calculations, all empty β-CD and all complex conformers of β-CD/benzene were thermodynamically allowed, i.e., the complexes should be formed. The highest relative energy of all conformers was less than 2.4 kcal/mol^−1^ in vacuo and less than 6.4 kcal mol^−1^ in aquo. The benzene molecule adopted an oblique position in the inclusion complex. The σ-surface of the empty β-CD “looked” more hydrophilic from the outside at its O2/O3 side (red and blue) than from the O6 side (only red and yellow/green). From the inside, the O6 rim of β-CD “looked” hydrophobic.

### Molecular-dynamics simulations

#### The empty cyclodextrin at 300 K

**Figure 8:**
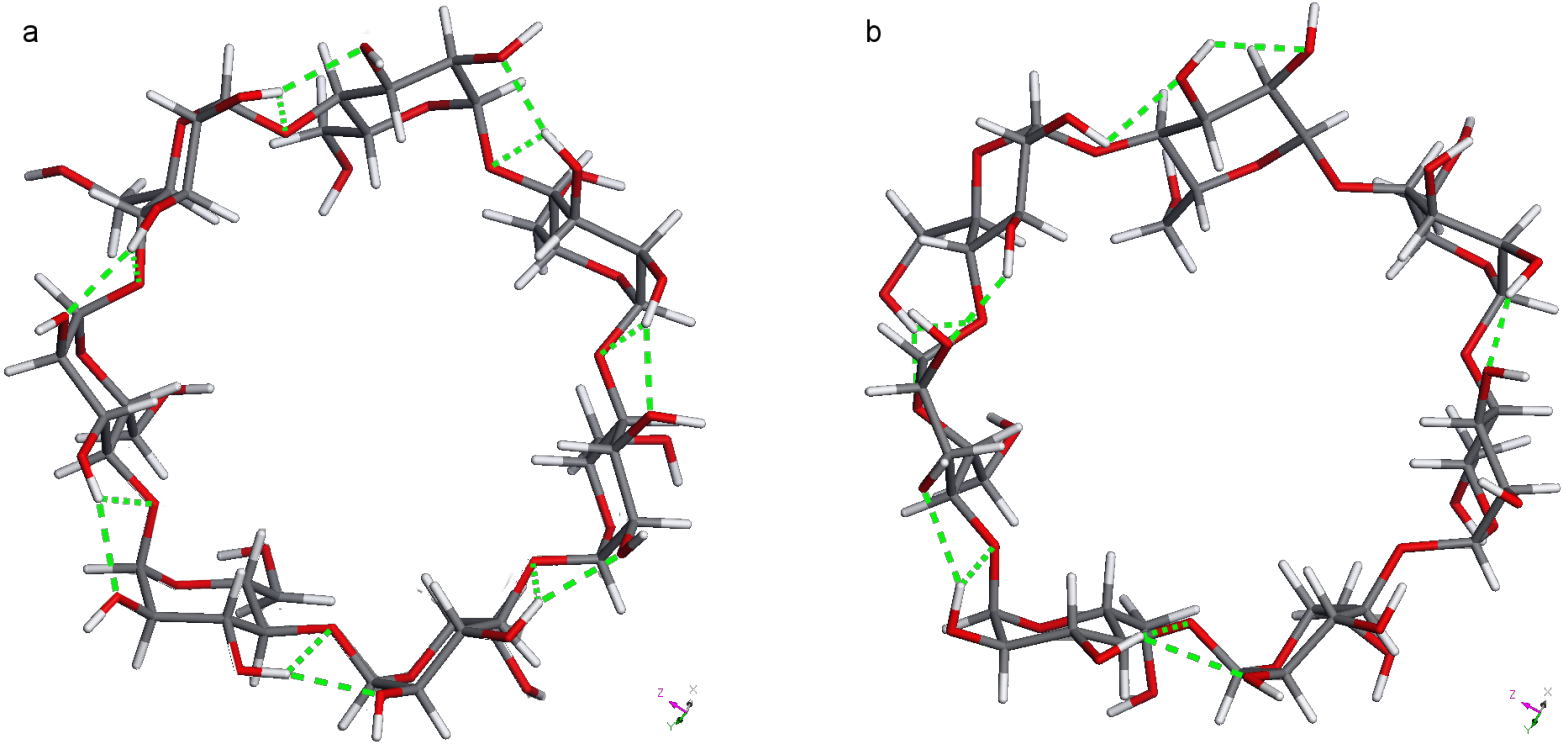
a: BCDcry (MD starting structure with Compass-*E*_tot_ = 389.99 kcal mol^−1^ and 14 hydrogen bonds with averaged Dreiding-*E*_HB_ = −3.04 kcal mol^−1^ => −42.56 kcal mol^−1^) and b: BCDcry fr1662 (best frame from the last 2000 ps of the analysed MD-trajectory with Compass-*E*_tot_ = 308.01 kcal mol^−1^ and 11 hydrogen bonds with averaged Dreiding-*E*_HB_ = −0.93 kcal mol^−1^ => −10.23 kcal mol^−1^).

**Figure 9:**
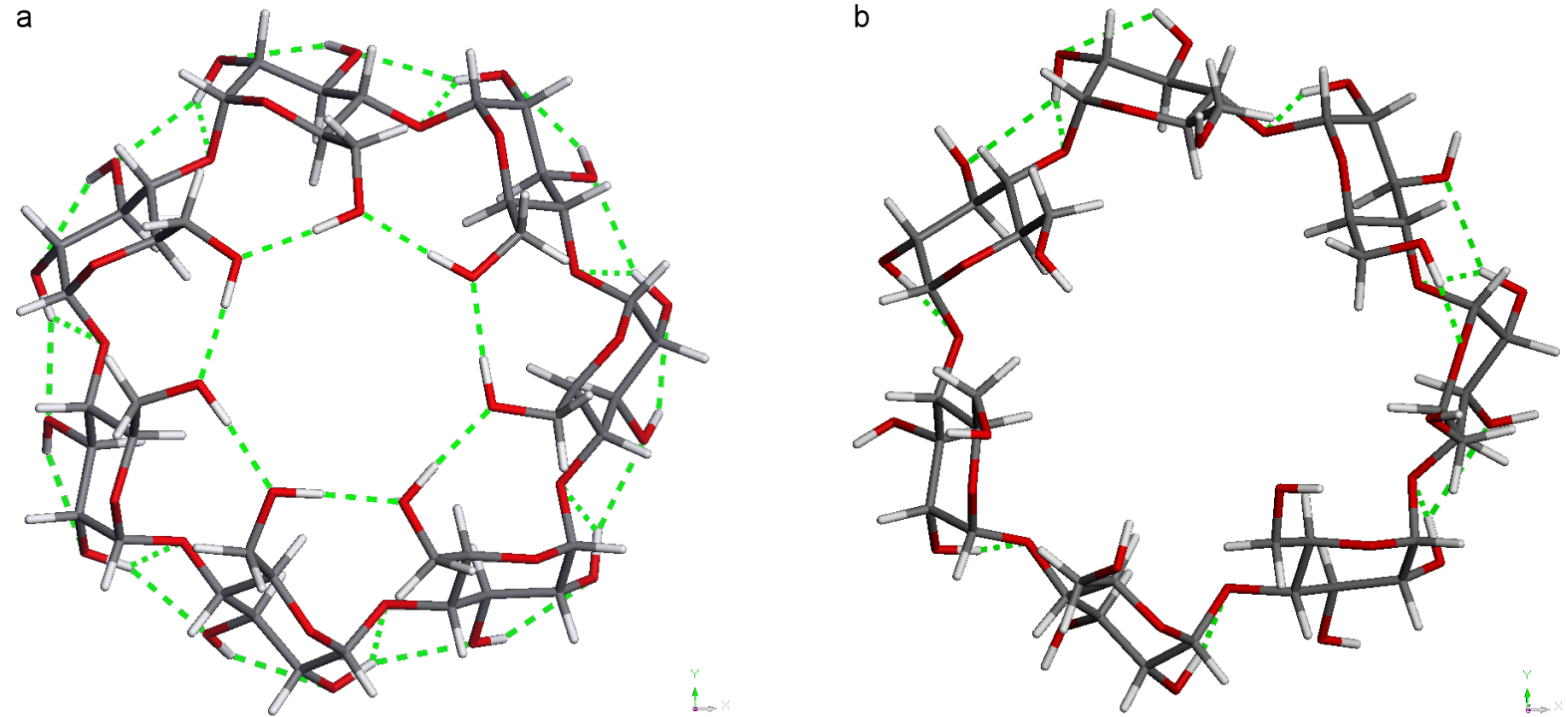
a: BCDO23rO6l (MD starting structure with Compass-*E*_tot_ = 221.52 kcal mol^−1^ and 28 hydrogen bonds with averaged Dreiding-*E*_HB_ = −4.71 kcal mol^−1^ => −131.88 kcal mol^−1^) and b: BCDO23rO6l fr4739 (best frame from the last 2000 ps of the analysed MD-trajectory with Compass-*E*_tot_ = 292.47 kcal mol^−1^ and 13 hydrogen bonds with averaged Dreiding-*E*_HB_ = −0.63 kcal mol^−1^ => −8.19 kcal mol^−1^).

**Figure 10:**
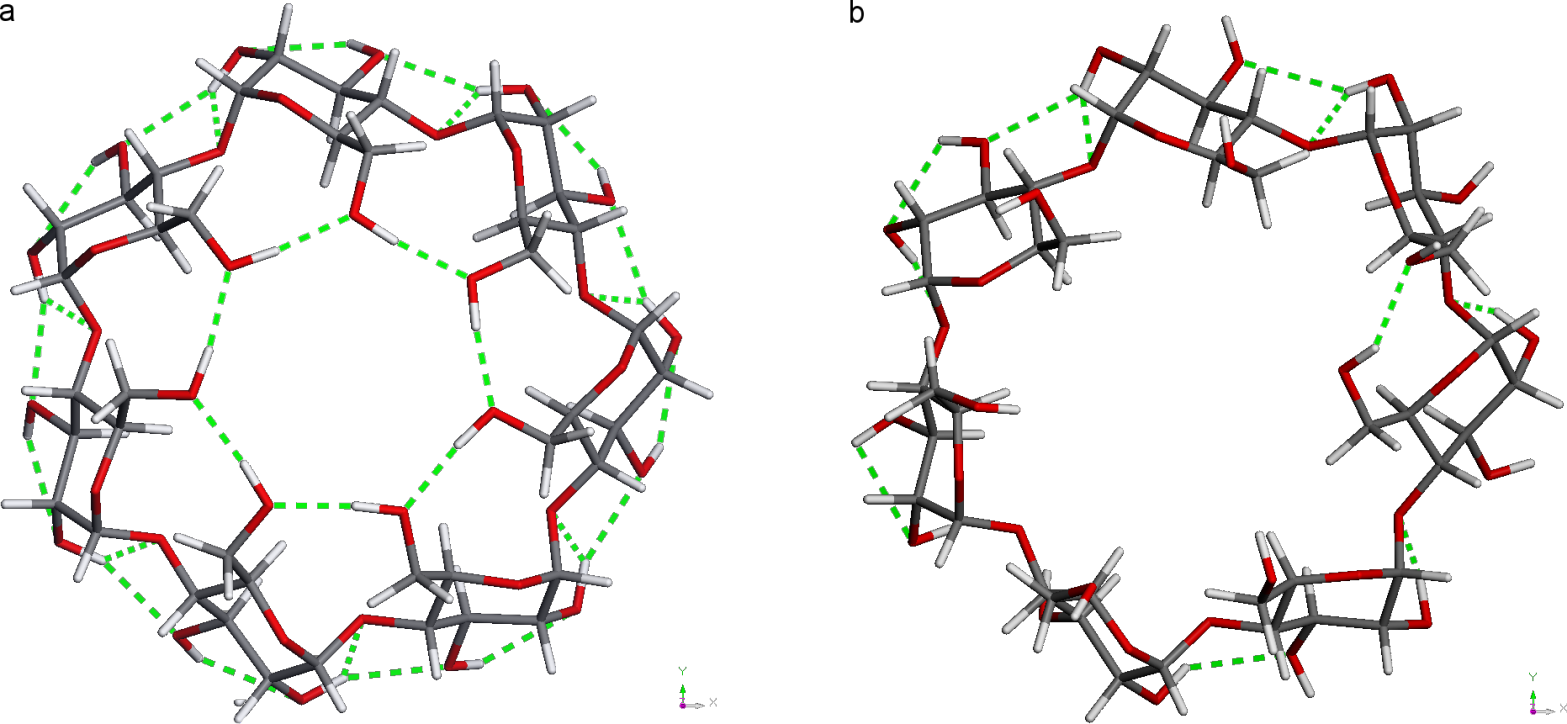
a: BCDO23rO6r (MD starting structure with Compass-*E*_tot_ = 217.25 kcal mol^−1^ and 28 hydrogen bonds with averaged Dreiding-*E*_HB_ = −4.59 kcal mol^−1^ => −128.52 kcal mol^−1^) and b: BCDO23rO6r fr5418 (best frame from the last 2000 ps of the analysed MD-trajectory with Compass-*E*_tot_ = 290.26 kcal mol^−1^ and 14 hydrogen bonds with averaged Dreiding-*E*_HB_ = −0.65 kcal mol^−1^ => −9.1 kcal mol^−1^).

The “highly ordered hydrogen bonds” of the BCDO23lO6l, BCDO23rO6l, BCDO23lO6r and BCDO23rO6r models cannot be observed at 300 K where the temperature is high enough to make the β-CDs flexible and the energy barriers of hydrogen bonds are overcome. As was observed in the neutron diffraction studies of β-CDs at 300 K [33] and 120 K [35] the temperature determined the hydrogen-bond distribution. In the MD simulations started from the “highly ordered” 0 K AM1 models the hydrogen bonds switched to more disordered arrangements. For comparison the crystal structure model of 300 K was added. The starting structures and the energetically best frame from the last 2000 ps of the 6000 ps trajectories are displayed in Figure 8, Figure 9 and Figure 10. Only the two energetically best models are shown: BCDO23rO6l and BCDO23rO6r. The Compass-*E*_tot_ energy of BCDcry was higher in the starting structure than in the best frame of the trajectory at 300 K, which accounts for the number of packing forces in the crystal that deformed the cyclodextrin monomer in vacuo. For the two other models BCDO23rO6l and BCDO23rO6r this was reversed; their “highly ordered hydrogen-bond structures” in vacuo from the start were energetically lower than their structures from their best frames of the trajectories at 300 K, because they “lost their hydrogen-bond stabilisation” and became flexible instead. All starting structures had much higher hydrogen-bond energies (analysis using Dreiding force field) than the best structures from their trajectories.

The five MD trajectories of the empty β-CD models had the following averaged energies (Compass force field): BCDcry 311.15 kcal mol^−1^, BCDO23lO6l 304.26 kcal mol^−1^, BCDO23lO6r 304.02 kcal mol^−1^, BCDO23rO6l 295.14 kcal mol^−1^ and BCDO23rO6r 291.22 kcal mol^−1^. According to this the BCDO23rO6r model was the lowest energy conformer, closely followed by the BCDO23rO6l model, which was the best conformer in the AM1 method. This means, that the hydrogen bonds at the larger O2/O3 rim of empty β-CDs preferred the right-hand orientation, i.e., O3–H^…^O2–H in the same glucose unit and bifurcated towards O4 and O3 of the next glucose unit on the right side.

#### The β-CD/benzene complex at different temperatures from 300 K to 120 K

**Figure 11:**
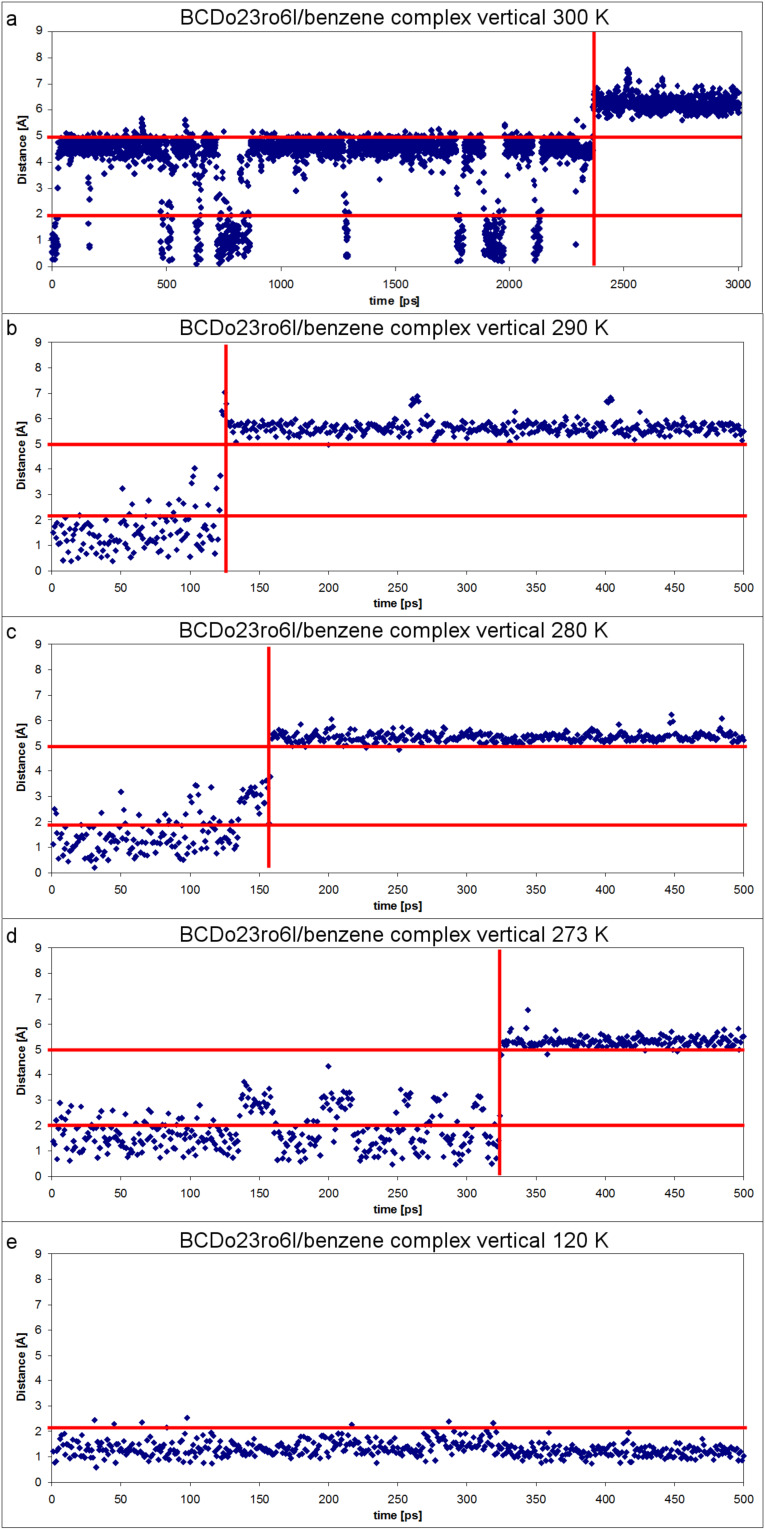
Distance plots from the MD trajectories of 500 ps each (*x* axis) for β-CD/benzene complex at different temperatures: a: 300 K, b: 290 K, c: 280 K, d: 273 K and e: 120 K. Distance [Å] (*y* axis) between the centres of mass of benzene and the seven O4 oxygen atoms of β-CD; horizontal red lines at distances of 2 Å and 5 Å, see text; vertical red lines indicate the time when the benzene left the β-CD cavity without return.

**Figure 12:**
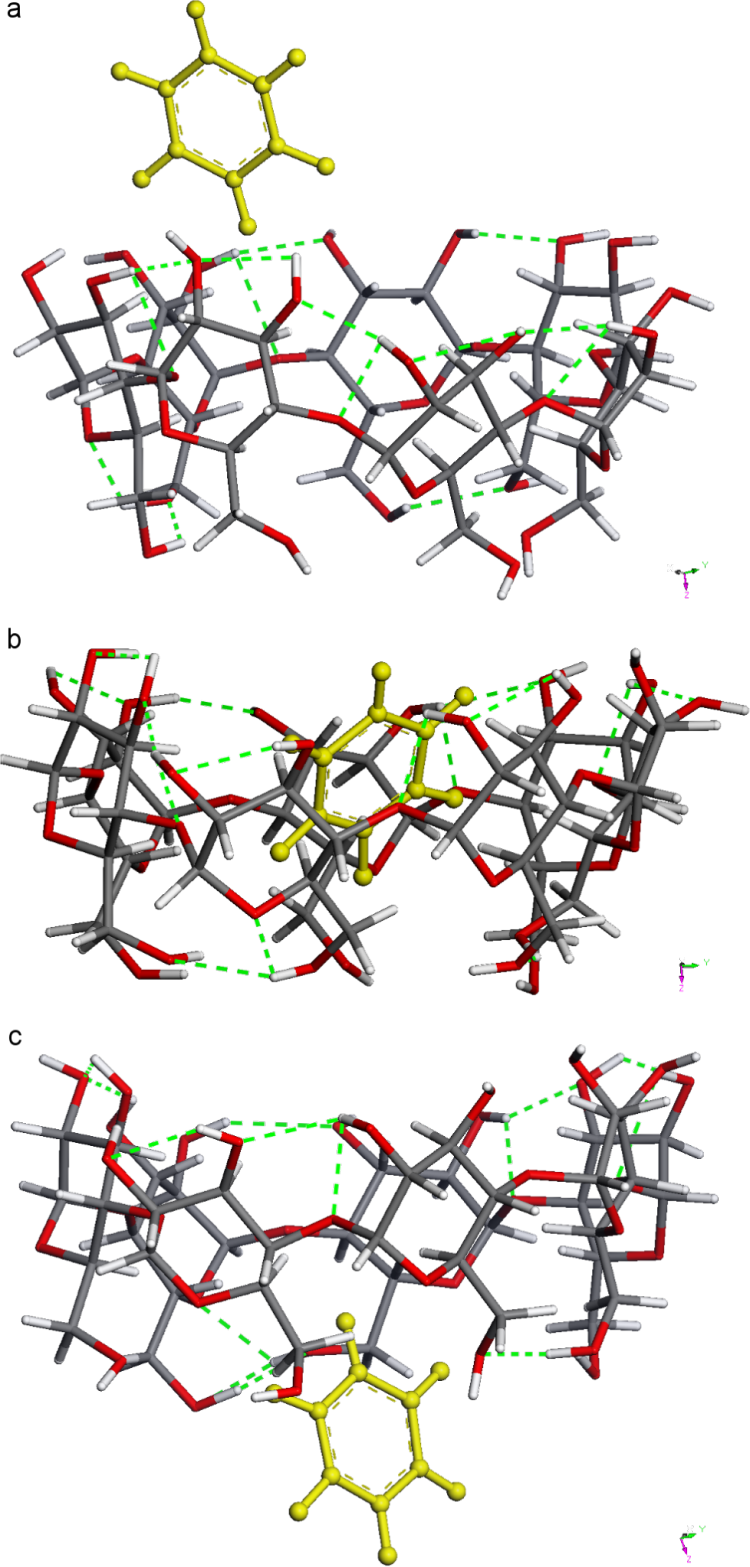
Three structures from the MD trajectory at 300 K: a: benzene left the β-CD at the O23 side; b: β-CD/benzene complex; c: benzene tried to leave the β-CD at the O6 side early, but always returned. Benzene finally left the cyclodextrin cavity also at the O23 side.

**Figure 13:**
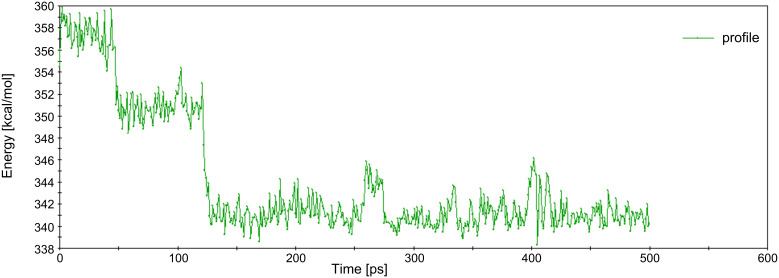
β-CD/benzene complex energy diagram (Forcite analysis – Hamiltonian) of the MD trajectory at 290 K.

The MD trajectories of the parallel and vertical BCDO23rO6l model were practically the same since the benzene guest changed its position already in the first few pico seconds, and later a significant difference could not be observed any more. It was observed that the benzene guest left the cyclodextrin host at slightly different times, depending on the temperature, (Figure 11). At 120 K benzene was always inside the cyclodextrin cavity. “Inside the cavity” was defined by a distance of up to 2 Å between the centre of mass of benzene and the seven O4 oxygen atoms of the β-CD that define a plane in the middle of the torus. At 273 K this distance extended up to 3 to 4 Å starting at about 140 ps, and at about 320 ps it became longer than 5 Å indicating that now benzene had left the cavity without return. At 280 K and 290 K these events appeared earlier, at 140 ps/160 ps and 50 ps/125 ps, respectively. This was in good qualitatively agreement with the complex formation constants that were experimentally determined from UV spectra in [34] and showed that the complex was more stable at lower temperatures. At 300 K benzene tried to leave the cavity early at the O6 side, but always returned. It spent most of the time at a greater distance of about 4 to 5 Å to the O4-plane of β-CD and finally left the cavity on the O2/O3 side as all the others did, but later at 2400 ps. Benzene had a high mobility in many geometrical positions in these trajectories, and the most characteristic ones from the trajectory at 300 K are shown in Figure 12. Figure 13 displays how different the energies of the β-CD/benzene complex were during the MD trajectory at 290 K.

#### Summarised result of the molecular dynamics simulations

Molecular dynamics simulations at different temperatures (120 K to 300 K) displayed qualitatively correctly the dynamics of the β-CD/benzene complex. Benzene accepted many different positions inside the β-CD cavity before it finally left at the O2/O3 side. The highly ordered hydrogen bonds at the O6 or O2/O3 rim were not existent at these temperatures and thus were a phenomenon of very low temperatures.

Since quantum-mechanical calculations lead to these highly ordered optimised structures, they represent the lowest energy conformers in vacuo at 0 K. Nevertheless, they will be the basis for the assumptions of the COSMO-RS method and its thermodynamic formulas and calculations/extrapolations based on the energetically lowest single-molecular states, plus conformers on top of that, up to 10 kcal mol^−1^. These conformer ensembles will make it possible to calculate several thermodynamic properties at room temperature and above, not only for the gas phase but also for the liquid and solid states of matter made from these molecules.

## Conclusion

The β-CD/benzene AM1 energies of complex formation after geometry optimisations for both positions of the guest, parallel and vertical were −2.75 kcal mol^−1^ and −2.46 kcal mol^−1^, respectively. The concerted highly ordered hydrogen bonds at the O2/O3 and O6 rims of cyclodextrin had a strong influence on the structures. This was confirmed by the calculated AM1 IR spectra, which showed that the β-CDs O–H frequencies were mostly split up upon complex formation with benzene. The HOMO/LUMO gap of the empty cyclodextrin with about 12 eV was lowered to about 10 eV in the complex, in agreement with other calculations (PM3) from the literature. All four hydrogen-bond models studied here (BCDO23lO6l, BCDO23rO6l, BCDO23lO6r and BCDO23rO6r) were energetically advantageous after geometry optimisations with BP86/TZVP-DISP3 in vacuo and in aquo, their relative energies were, all but one, less than 3.2 kcal mol^−1^. The character of the COSMO-RS σ-surface of β-CD was much more hydrophobic on its O6 rim than on its O23 side when all hydrogen bonds were arranged in a concerted mode. COSMO-RS BP/TZVP-DISP3 calculations in vacuo and in aquo (dielectric field approximation) preferred an oblique position (over parallel or vertical) of benzene inside the β-CD cavity and suggested energies of complex formation up to 20.2 kcal mol^−1^ in vacuo and of about 16 kcal/mol^−1^ in aquo. The static picture of these energetically lowest structures of the β-CD/benzene complex at 0 K was extended by the molecular dynamics simulations at different temperatures (120 K to 300 K). They displayed qualitatively correctly the dynamics of the β-CD/benzene complex, which was more stable at lower temperatures, especially at 120 K. Benzene accepted many different positions inside the β-CD cavity before it finally left at the O2/O3 side (273 to 300 K). The highly ordered hydrogen bonds at the O6 or O2/O3 rim were a subtle phenomenon at very low temperatures. Still, we assume that hydrogen bonds of cyclodextrins nevertheless play a crucial role in all their special properties, i.e., their many inclusion complexes and remarkable influence on reactions, catalysis and supramolecular structures.

## References

[1] Szejtli J (1998). Chem Rev.

[2] Jeffrey G A, Saenger W (1991). Chapter 4. Theoretical calculations of hydrogen bond geometries. Hydrogen Bonding in Biological Structures.

[3] Alderfer J L, Eliseev A V (1997). J Org Chem.

[4] Schmid B V K J, Hetzer M, Ritter H, Barner-Kowollik C (2011). Macromolecules.

[5] Eliadou K, Yannakopoulou K, Rontoyianni A, Mavridis I M (1999). J Org Chem.

[6] Aachmann F L, Otzen D E, Larsen K L, Wimmer R (2003). Protein Eng, Des Sel.

[7] Willerich I, Schindler T, Ritter H, Gröhn F (2011). Soft Matter.

[8] Park C, Oh K, Lee S C, Kim C (2007). Angew Chem, Int Ed.

[9] Kida T, Iwamoto T, Fujino Y, Tohnai N, Miyata M, Akashi M (2011). Org Lett.

[10] Koehler J E H, Saenger W, van Gunsteren W F (1988). J Biomol Struct Dyn.

[11] Köhler J, Hohla M, Söllner R, Eberle H-J (1998). Supramol Sci.

[12] Köhler J, Hohla M, Söllner R, Amann M (1998). Supramol Sci.

[13] Yu H, Amann M, Hansson T, Köhler J, Wich G, van Gunsteren W F (2004). Carbohydr Res.

[14] Damodaran K V, Banba S, Brooks C L (2001). J Phys Chem B.

[15] Liu L, Guo Q-X (1999). J Phys Chem B.

[16] Liu L, Guo Q-X (1999). J Chem Inf Comput Sci.

[17] Liu L, Guo Q-X (2004). J Inclusion Phenom Macrocyclic Chem.

[18] Klamt A (2005). COSMO-RS From Quantum Chemistry to Fluid Phase Thermodynamics and Drug Design.

[19] Klamt A, Eckert F, Arlt W (2010). Annu Rev Chem Biomol Eng.

[20] Klamt A, Reinisch J, Eckert F, Hellweg A, Diedenhofen M (2012). Phys Chem Chem Phys.

[21] Saenger W (1980). Angew Chem.

[22] Ho B T, Joyce D C, Bhandari B R (2011). Food Chem.

[23] Regiert M (2009). SOFW J.

[24] Schmid G, Atwood J, Davies E D, MacNicol D D (1996). Preparation and industrial production of cyclodextrins. Comprehensive Supermolecular Chemistry.

[25] van der Veen B A, Uitdehaag J C M, Dijkstra B W, Dijkhuizen L (2000). Biochim Biophys Acta.

[26] Li Z, Wang M, Wang F, Gu Z, Du G, Wu J, Chen J (2007). Appl Microbiol Biotechnol.

[27] Rungsardthong Ruktanonchai U, Srinuanchai W, Saesoo S, Sramala I, Puttipipatkhachorn S, Soottitantawat A (2011). Biosci, Biotechnol, Biochem.

[28] Hobza P, Selzle H L, Schlag E W (1994). J Am Chem Soc.

[29] Materials Studio.

[30] (2009). Gaussian 09.

[31] Turbomole.

[32] Grimme S, Antony J, Ehrlich S, Krieg H (2010). J Chem Phys.

[33] Betzel C, Saenger W, Hingerty B E, Brown G M (1984). J Am Chem Soc.

[34] Trofymchuk I M, Belyakova L A, Grebenyuk A G (2011). J Inclusion Phenom Macrocyclic Chem.

[35] Zabel V, Saenger W, Mason S A (1986). J Am Chem Soc.

